# Estimation of haemoglobin using non-invasive portable device with spectroscopic signal application

**DOI:** 10.1038/s41598-024-58990-z

**Published:** 2024-04-15

**Authors:** A. M. Amrutha, Bhagyalaxmi Sidenur, Balu P.S, Savitha S.V, Nagendra Gowda M.R, Harshavardhan Rajagopal

**Affiliations:** 1 Department of Community Medicine, Basaveshwara Medical College and Hospital, Chitradurga, India; 2 C-DART, Basaveshwara Medical College and Hospital, Chitradurga, India; 3 SJM Institute of Nursing, Chitradurga, India; 4 American Hospital Dubai, Dubai, UAE

**Keywords:** Anaemia, Nursing students, Non-invasive, Spectroscopy, EzeCheck, Haemoglobinometer, Health care, Medical research

## Abstract

An estimated 52% of non-pregnant women of reproductive age in India are estimated to be affected by anaemia, which is categorised as a chronic condition. In 2019–2021, the National Family Health Survey–5 (NFHS–5) which was undertaken revealed the following statistics about the prevalence of anaemia in the state of Karnataka. To estimate haemoglobin levels using non-invasive portable device among nursing students. A cross sectional study was done among students of Nursing college in central Karnataka for a period of 3 months. Total of 140 students were included in the study. EzeCheck haemoglobin estimation was done twice and was recorded in the same Google form. The frequency and percentage of variation of results between Haematology Analyzer and EzeCheck devices was presented with a range of difference such as 0, less than 1, 1.0 to 1.9, 2.0 to 2.9, 3.0 to 3.9, and 4.0 and above. The total prevalence of anaemia among nursing students was 57.8% and most of the students had moderate degree of anaemia (28.6%). Two readings of haemoglobin were taken and difference of readings were calculated and majority of the students had difference of < 0.5 gm/dl (61.4%) and only 1.4% of the students had difference of > 2 gm/dl. The mean difference of haemoglobin of two readings was 0.5 ± 0.5 gm/dl**.** The technology employed in this study bridges the gap between patients and anaemia diagnosis by providing screening services. The device provides the diagnosis via a non-invasive, IoT-enabled service at a low cost.

## Introduction

In India, around 52% of non-pregnant women of reproductive age, are estimated to be affected by anaemia, which is categorised as a chronic condition^1^. Anaemia, which is caused by a lack of haemoglobin, has an impact on how well our organs work. Numerous factors contribute to anaemia, including decreased red blood cell synthesis, blood loss from numerous causes and accelerated red blood cell breakdown owing to haemolysis^2^. Most anaemic women and teenagers experience gynaecological issues, tiredness, and slowed cognitive function. They also tend to have slow growth.

People are unaware of the basic function and importance of haemoglobin in the human body. There is a delay in diagnosing anaemia due to a lack of diagnostic technologies for adequate screening and expensive clinical tests for detecting haemoglobin deficiency. This leads to delayed procedure between screening and providing medical assistance^3^.

In 2019–2021, the National Family Health Survey–5 (NFHS–5) which was undertaken revealed the following statistics about the prevalence of anaemia in the state of Karnataka: Among women aged 15–49 years: 43.9% in urban areas and 50.3% in rural areas, while 62.8% in urban areas and 67.1% in rural areas among children aged 6–59 months (NFHS 5, Karnataka Fact Sheet)^4^. These figures show that anaemia is a significant public health issue in urban and rural Karnataka, with rural women and children experiencing higher rates.

The main causes of anaemia in Karnataka are like those in other parts of India, including inadequate dietary intake of iron and other essential nutrients, poor absorption of iron due to parasitic infections, and chronic blood loss due to menstruation and childbirth^5^.

Due to inadequate hygienic conditions of the surroundings and medical appliances, the majority of patients suffer from infections after undergoing haemoglobin screening at hospitals/clinics; there is a tremendous demand for non-invasive devices.

Nursing students are an essential component of our healthcare system because they will be the future nurses and primary healthcare providers^6^. They should first be aware of the nutritional difficulties they face and the prevalence of anaemia within their own ranks so that, when they offer nursing care to the community, they can inform the population about the high prevalence rate of anaemia and the actions that can be taken to lower it. Hence, the present study was conducted to estimate haemoglobin levels using non-invasive portable device among nursing students.

## Methods

A Cross sectional study was conducted at a Nursing Medical College, Karnataka, India, between the period of June to August 2023. Appropriate approval and clearance from the institutional ethics committee (Basaveshwara Medical College and Hospital, Chitradurga) was obtained prior conducting this study.

Considering the prevalence of anaemia to be 44%, among nursing students according to Srivastav et.al.^6^, with 95% confidence level and 20% relative error, the calculated sample size was 126 which was inflated to 140 for statistical convenience. The sample size was calculated using estimation technique: N = Z^2^PQ/d^2^.

All the Nursing students who provided their consent to participate in this study, were included in our study. Students who had cut on their left-hand ring finger, or had excess sweat or had skin peeling or redness on the left-hand ring fingertip were excluded from this study as it could interfere with the results. Although in our study, no enrolled participants had applied mehndi on their fingertip, but as per the manufacturer’s instructions, it is advised to exclude those participants as well who have applied heena/mehndi on their left-hand ring fingertip.

Appropriate written informed consent was taken from all the participants who were willing to participate in this study, and was recorded in the Google form.

**Figure 1:**
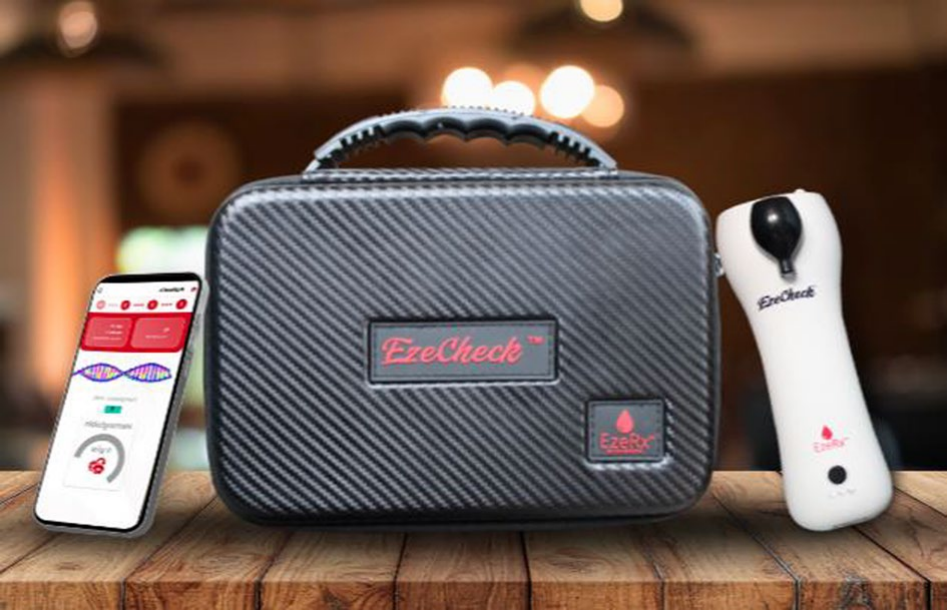
EzeCheck product image.

EzeCheck haemoglobin estimation was done twice and was recorded in the same Google form. The frequency and percentage of results recorded with EzeCheck device (Fig. 1) were presented with a range of difference such as 0, less than 1, 1.0 to 1.9, 2.0 to 2.9, 3.0 to 3.9, and 4.0 and above.

The entire data collection process was carried out by following appropriate ethical committee’s guidelines to ensure data privacy of the participants.

Characteristics of EzeCheck^7^:

EzeRx has created a device named EzeCheck, that analyses a person's blood for anaemia and haemoglobin levels. The device offers simple ways to encourage routine health examination and raise awareness of anaemia in the general public. Since, the device doesn't need any medical equipment or human blood to estimate the haemoglobin levels, so the testing can be performed without any assistance from the medical expert, making it accessible to the general public. Haemoglobin screening using the EzeCheck device is entirely painless and produces digital reports in a matter of minutes, which speeds up the medical procedure, resulting in better cure and prevention of illnesses.

**Figure 2:**
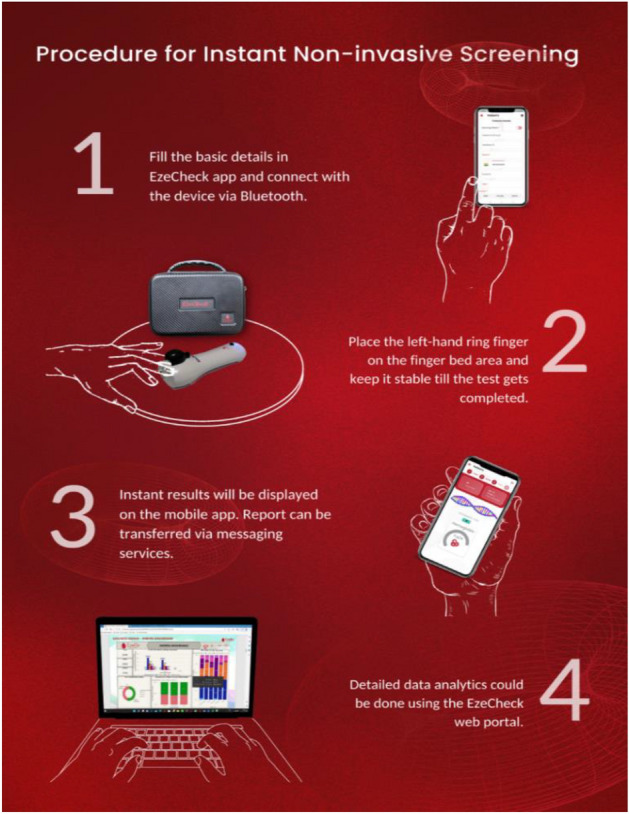
Representation of the process involved for conducting the Non-invasive Haemoglobin test using the EzeCheck device.

A simplified procedure for obtaining the Hb results using the EzeCheck device is being represented in the Fig. 2.

Protocol for EzeCheck^7^^:^
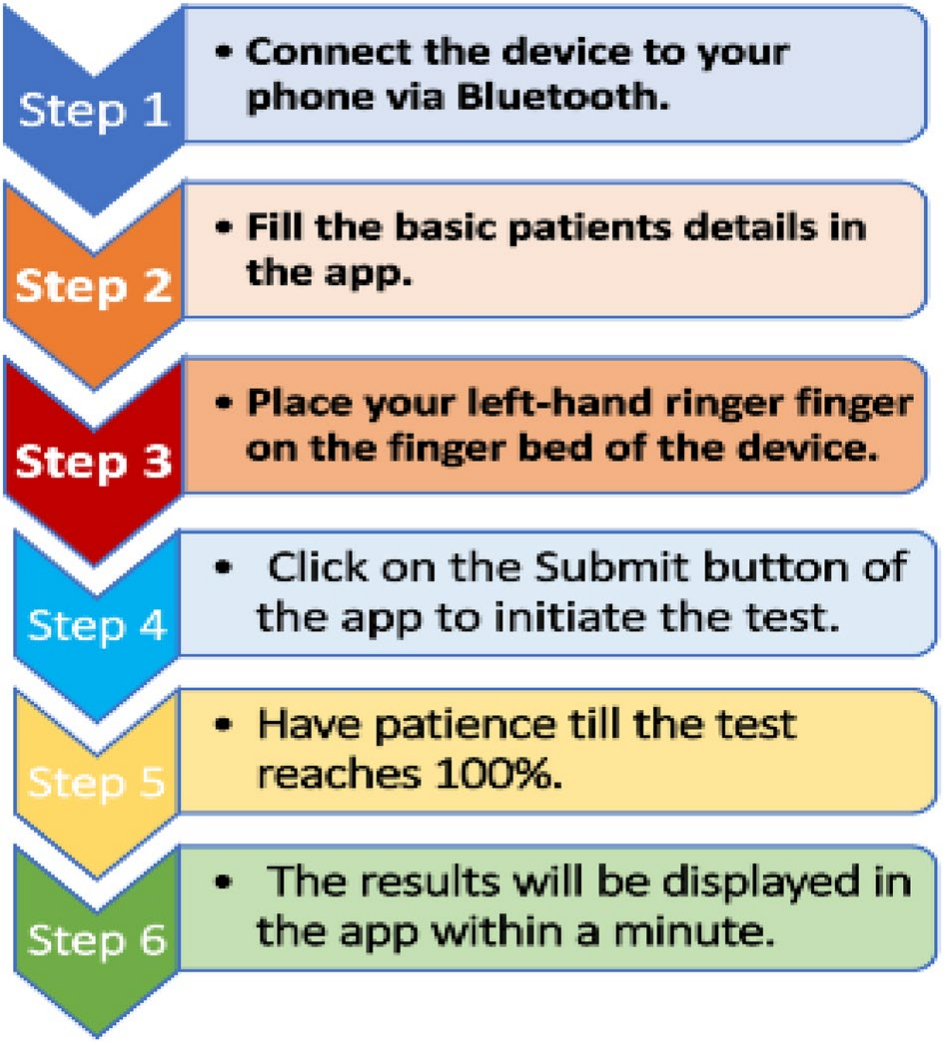


### Statistical analysis

The data collected was entered into an MS excel spread sheet and analyzed using IBM SPSS version 20.0 software. Socio-demographic data was presented using descriptive statistics in the form of mean/proportion, median, standard deviation and percentage whichever was applicable. Chi-square test was applied to test the association of various variables to the severity of anemia.

### Ethical approval

Prior Ethical Approval was obtained from the Institutional Committee (Basaveshwara Medical College and Hospital, Ref. BMC&H/IEC/152/2023-24). The study was conducted in accordance with the Helsinki Declaration. Data of all the individuals are kept strictly confidential to ensure data safety and privacy.

## Results

A total of 140 nursing students participated in the study. The study population included 65.7% of females and 34.3% of males and majority of the students belonged to 18–20 years (56.4%) of age with mean age of 20.5 ± 1.6 years (Table 1).

**Table 1:** Demographic characteristics.

Variables	Frequency	Percentage
Age (in years)		
18–20	79	56.4%
21–23	53	37.9%
24–26	7	5%
> 26	1	0.7%
Gender		
Female	92	65.7%
Male	48	34.3%
Place of residence		
Rural	59	42.1%
Urban	81	57.9%

The total prevalence of anaemia among nursing students was 57.8% and most of the students had moderate degree of anaemia (28.6%). Compared to men (10%), females (47.8%) had a higher prevalence of anaemia (Table 2).

**Table 2:** Prevalence of anaemia among nursing students.

Anaemia	Frequency	Percentage
Normal	59	42.1%
Mild	33	23.6%
Moderate	40	28.6%
Severe	8	5.7%
Total	140	100%

In our study, two readings of haemoglobin were taken, and difference of readings were calculated, and majority of the students had difference of < 0.5 gm/dl (61.4%) and only 1.4% of the students had difference of > 2 gm/dl. The mean difference of haemoglobin of two readings was 0.5 ± 0.5 gm/dl (Table 3).

**Table 3:** Difference of Hb of two readings.

Difference of Hb	Frequency	Percentage
< 0.5	86	61.4%
0.5–1	32	22.9%
1.1–1.5	10	7.1%
1.6–2	10	7.1%
> 2	2	1.4%
Total	140	100%

Our study revealed that majority of the students in the age group 18–20 years had mild (54.5%), moderate (52.5%) and severe (62.5%) degrees of anaemia compared to other age groups. Most of the female nursing students had mild (69.7%), moderate (90%) and severe (100%) degrees of anaemia compared to males and the association was highly significant (Table 4).

**Table 4:** Association of variables with anaemia.

Variables	Grades of Anaemia				P value
	Normal	Mild	Moderate	Severe	
Age (in years)					
18–20	35 (59.3%)	18 (54.5%)	21 (52.5%)	5 (62.5%)	0.8
21–23	20 (33.9%)	13 (39.4%)	17 (42.5%)	3 (37.5%)	
24–26	4 (6.8%)	2 (6.1%)	1 (2.5%)	0	
> 26	0	0	1 (2.5%)	0	
Gender					
Female	25 (42.4%)	23 (69.7%)	36 (90%)	8 (100%)	< 0.001
Male	34 (57.6%)	10 (30.3%)	4 (10%)	0	

In our study, 89.4% of the students with normal Hb in the first reading showed normal levels in the second reading. About 54.5% of the students with mild anaemia in the first reading also showed mild in the second reading and 65.7% of the students with moderate anaemia in the first reading also showed moderate in the second reading. About 83.3% of the students with severe anaemia also showed severe in the second reading and the association of classification of anaemia between first and second reading was highly significant (Table 5).

**Table 5:** Classification of haemoglobin in two consecutive readings.

First Hb reading	Second Hb reading				*P* value
	normal	Mild anaemia	Moderate anaemia	Severe anaemia	
Normal	59 (89.4%)	6 (9.1%)	1 (1.5%)	0	** < 0.001**
Mild anaemia	9 (27.3%)	18 (54.5%)	6 (18.2%)	0	
Moderate anaemia	2 (5.7%)	8 (22.9%)	23 (65.7%)	2 (5.7%)	
Severe anaemia	0	0	1 (16.7%)	5 (83.3%)	

Significant are in value [bold].

## Discussion

In India, nutritional anaemia, which is mostly brought on by a lack of iron, is a significant public health issue. Anaemia is prevalent across all age groups and is more prevalent in the most vulnerable populations, where it affects 50% of non-pregnant non-lactating women and nearly 58% of pregnant women, according to the National Family Health Survey-3 data (NFHS-3)^8^.

Anaemia has detrimental effects on both social and economic progress and human health. One million fatalities per year are attributed to anaemia, the second-leading cause of disability in the world, with the majority of these deaths occurring in Southeast Asia and Africa^9^.

The physical and mental disabilities caused by IDA result in annual GDP losses of up to 4.05 percent, halting social and economic growth. These losses amount to 1.18 percent of India's GDP when results are reported as a percentage of GDP^10^.

In our study, the total prevalence of anaemia among nursing students was 57.8%. In the study done by Shrivastav et al., the overall prevalence rate was 44% and another study done by Khan et al.^11^ where total prevalence was 39% prevalence. Similarly, a study done in North East Delhi^12^ reported a prevalence of anaemia as 45%. Chandrakumari et al.^13^, however, found a prevalence of 48.63% overall. The fact that most students were living in hostels, which were far from their homes, may have contributed to the high prevalence. Students who live in hostels frequently do not adhere to a rigorous diet, and they may detest the quality of the food made and served there, leading them to order food from a restaurant. As a result, more junk food is consumed and meals are skipped.

Our study showed that compared to men (10%), females (47.8%) had a higher prevalence of anaemia. which is similar to the studies done by Shrivastav et al.^14^ (39%) and Khan at al.^11^ (56%) where prevalence with preponderance among female nursing students was seen. The additional requirement for iron caused by menstrual blood loss, malnutrition brought on by dietary changes, insufficient consumption of foods high in iron, or being on a diet may be the cause of the gender imbalance^11,15,16^. On the other hand, men may be at risk for anaemia in situations when there is malnutrition as a result of insufficient dietary intake.

Our study reported that majority of the students were 18–20 years of old (56.4%) and they (31.4%) had high prevalence of anaemia compared to more than 20 years old (26.4%). Majority of the students had moderate degree of anaemia (28.6%). In the study done by MC Yadavannavar et al.^17^ also showed high prevalence of anaemia among 18–20 years of age (34.6%). This is a difficult age for students because they are moving away from their homes for higher studies and must adjust to life in a dormitory. Medical and nursing students are more likely to acquire anaemia as a result of irregular eating habits caused by a demanding academic schedule^18^. They are at risk of anaemia due to variables like as living in dorms, skipping meals, clinical rotations, hectic schedules at college and hospital, and others^19^.

Hb estimation via an invasive approach is not an ideal solution, especially for those living in remote places with limited access to medical care. This intrusive approach also has a number of drawbacks, such as the fact that diagnostic tools are not portable, results are not always accessible right away, and the entire process is expensive. Every year, more than a million people die as a consequence of delayed diagnosis of serious health issues and more than a lac people die as a result of delayed diagnosis of anaemia. These various instances show how important a non-invasive approach for measuring haemoglobin is^20,21^.

There is already a tonne of commercially available non-invasive POC tools for measuring haemoglobin, but all of them have one or more flaws, like challenging data collection methods, challenging data analysis and feature extraction processes, lacking portability and affordability, and having unfriendly user interfaces with pricey external modules. Smartphone-based solutions are getting more and more common because of all of its advantages. Recent Hb level measurements use signal gathered from parts of the human body like the fingertip, nail beds, and lower eyelid. The smartphone's built-in sensors, extra attachments, signal processing methods, and machine learning algorithms all significantly improve hb level estimation^22^. Although minimally invasive devices like HemoCue have reduced the sample processing time and the cost for testing but still is a painful method for some individuals and children as it involves pricking the fingertip. A study conducted on blood donors, to determine the efficacy of a non-invasive Hb meter (OrSense NBM 200) had revealed that the non-invasive technology can be a great help towards prick-free and zero biomedical waste generation process for estimating the Hb levels^23^. Although, the results are promising but the technology has certain limitations such as no data traceability due to lack to digital record keeping system, also the device is not handy as it is a table top device which is entangled with few wires as well, making it difficult to carry everywhere. Unlike this, the EzeCheck could be easily carried and doesn’t require any additional wire attachments for conducting the test. Also, the reports can be easily traced even after years, just by selecting the appropriate date of test conduction, from the records section of the mobile app.

Khalafallah et al. in their study had shown the significance of Pronto-7 pulse CO-oximetry device (Masimo Corp.) in determining the Hb levels for preoperative assessment. The observed results have although indicated values in the unacceptable range but have highlighted the significance of the non-invasive technologies as it will enable faster anaemia diagnosis and management at the time of initial clinic visit^24^. This will additionally reduce the travel and accommodation expenses of the patients who need to visit the next day to collect their reports, especially for those who resides in different rural areas and have to visit to a city-based hospital for proper treatment.

In our study, EzeRx, as a healthcare startup, aims to tackle this issue with EzeCheck -ICMR validated non-invasive portable medical device with Spectroscopic Signal Application that screens haemoglobin instantly^7^. The device takes total of 1 and a half minute (Starting from log in into the app to result display) to give the reading and in our study, two readings of haemoglobin were taken and difference of readings were calculated and majority of the students had difference of < 0.5 gm/dl (61.4%) and only 1.4% of the students had difference of > 2 gm/dl. The mean difference of haemoglobin of two readings was 0.5 ± 0.5 gm/dl.

Majority of the students in mild, moderate and severe category in the first reading remained in the same categories in the consequent second reading. Hence, the device showed consistency in the readings. The prevalence of anaemia using the device came to 57.2% which is similar to NFHS 5^4^ data (men-25%, Women-57%). Hence, the results demonstrate that it has a satisfactory measurement accuracy non-invasively.

As the device uses fingertip placement, the readings are not affected by nail polish with respect to consistency of the readings, which is the major advantage of the device followed by painless, increased acceptance by patients and reduced issues with poor follow-up, safety, biohazard disposal, and supply chain needs. Another point which adds to the advantage is the screening on mass level in the remote areas by community health workers. Also, the device can work on both offline and online conditions, which is a major boon for the rural areas with poor network connectivity.

Our innovative method, rather than replacing the existing intrusive method of detecting haemoglobin concentration, is intended to be used as a preliminary test for anaemia. Patients who received negative results or were on the borderline would be urged to return to the hospital for additional testing using a standard haemoglobin concentration measurement technique. Additionally, the proposed technology can be utilised to monitor haemoglobin concentration in real-time because it is painless and non-invasive.

## Conclusion

Haemoglobin is a significant health indicator because it is responsible for a wide range of human body functions and illnesses. By providing screening services, the technology used in this study bridges the gap between patients and anaemia diagnosis. The device provides a low-cost diagnosis via a non-invasive, IoT-enabled service. This, in turn, aids in screening enormous populations worldwide, regardless of socioeconomic level, gender, or place of residence, and giving them with early medical assistance to lead a healthy lifestyle.

## Data Availability

The datasets for this article are not publicly available due to concerns regarding the participant’s anonymity. Requests to access the dataset should be directed to the corresponding author.
